# Factors determining choice of delivery place among women of child bearing age in Dega Damot District, North West of Ethiopia: a community based cross- sectional study

**DOI:** 10.1186/s12884-016-1020-y

**Published:** 2016-08-17

**Authors:** AlemayehuSayih Belay, EndalewGemechu Sendo

**Affiliations:** 1 Department of Nursing, Mizan Tepi University, College of Health Sciences, Mizan Teferi, Ethiopia; 2 Department of Nursing and Midwifery, Addis Ababa University, College of Health Sciences, Addis Ababa, Ethiopia P.O. Box 1176, Ethiopia

**Keywords:** Institutional delivery, Women of reproductive age, Dega Damot District, Ethiopia

## Abstract

**Background:**

In the latest report of Ethiopia Demography and Health Survey (EDHS) 2011, the Maternal Mortality Ratio was estimated at 676/100,000 live births. Most of these deaths are preventable. Increasing the proportion of women who deliver in a health facility can be an important means in reducing maternal mortality in low-income settings including Ethiopia.

We aimed to identify factors determining choice of delivery place among child bearing age women.

**Method:**

A community based cross sectional survey was conducted in Dega Damot District from April- May, 2014. Mixed methods were employed in the study. Multistage sampling method was used. The primary outcome variable for this study was women who delivered their most recent baby in a health facility.

**Result:**

Three hundred sixty one women who gave birth in the past 1 year were included in the study. The mean age of the respondents was 30.9 [SD ±6.006]. One hundred seven (29.6 %) of the respondents were in the age range of 25–29 years. In our study, the proportion of women assisted by skilled health workers during institutional delivery was 89.1 % followed by Health extension workers (8.0 %). Most women (87.4 %) who did not deliver in health facilities were assisted by families, friends or neighbors followed by Health extension workers (7.2 %), and traditional birth attendants (5.4 %), respectively.

The qualitative data has described and gave an insight of the contributing factors that influence the women using the health institutions for delivery. These included: ANC attendance, Positive attitude of Health workers and complications during labor and delivery. The preference for a health facility delivery was largely due to the understanding that if complications occurred either during labor or delivery, this was the only place where they could be managed.

**Conclusion:**

The study revealed that women’s institutional delivery service utilization in the study area is low. Based on these findings, improving the utilization of health facility for delivery through educating women and health promotion have been recommended. This would help reduce the complications and dangers that often characterized home-based, unsupervised delivery.

## Background

Millennium Development Goal 5 (MDG 5) set a target of 75 % reduction in maternal mortality, from 400/100,000 live births to 100/100,000 between the 1990 baseline and 2015 [1]. Increasing the proportion of women who deliver in a health facility can be an important means in reducing maternal mortality in low-income settings. It is globally recognized that one of the main challenges to achieving the MDG 5 of a global reduction of maternal death by 75 % by 2015 is the low proportion of women who deliver with a skilled birth attendant [2].

In many low-income settings with a high burden of maternal deaths, few women use facilities for birth, often choosing a higher-risk birth at home, often without professional medical assistance [3]. Deliveries in health facilities can ensure that women are attended by skilled personnel and also link women to the referral system in the case of any complications.

A key strategy for reducing maternal morbidity and mortality is thus ensuring that every birth occurs with the assistance of skilled health personnel, meaning a medical doctor, nurse or midwife. Progress in raising the proportion of births delivered with skilled attendance has been modest over the course of the MDG time frame, reflecting lack of universal access to care [4].

According to Mini Ethiopian Health Demography Survey (MEDHS) 2014, 40 % of pregnant women who gave birth in the 5 years preceding the survey received antenatal care once from a skilled provider, that is, from a doctor, nurse, or midwife, for their most recent birth, 34 % from a nurse or midwife, and 6 % from a doctor. But only 32 % of women with a live birth in the 5 years before the survey made four or more ANC visits during the length of their pregnancy, a marked improvement from 19 % reported in the 2011 EDHS [5].

Nevertheless, *only 15 %* of births in Ethiopia are delivered at a health facility—14 % in a public facility and 1 % in a private facility [5].

As a signatory of Millennium Declaration 2000, Ethiopia is dedicated to improving skilled birth care and hence reducing maternal mortality. It targets for 75 % reduction of MMR from 880/100,000 in 1990 to 220/100,000 in 2015. However, Ethiopia is still far off from the MDG 5 [6]. Ethiopia was also the least achiever in at least 4 visits antenatal care coverage in Sub-Saharan Africa. To be more objective, the proportion of health facility delivery and antenatal care between 1995 and 2011 were in the range of 5–15 % and 10–19 %, respectively [5–7]. Thus, the low proportion of antenatal care compounded by the extremely low skilled person attended delivery might be some of the major reasons for the high maternal mortality persisting during the last decade (873 and 676 per 100,000 live births in 2000 and 2011, respectively) [7]. Our present study focused on West Gojjam Zone because its utilization of SBA services is lower compared to other regions of Ethiopia.

## Methods

### Study design and study setting

A community based cross-sectional study mixed methods was conducted in Daga Damot District from April 2014 to May 2014.

Daga Damot District is located 399 Km from the capital city of Addis Ababa. It is one of the Districts in the West Gojjam, Amhara Region, Ethiopia. It has an area of 65726.68 Km^2^ with an estimated total population of 152,343 according to the 2007 national census which was conducted by the Central Statistical Agency of Ethiopia (CSA). The estimated total number of women of reproductive age group and pregnant women in the district (both rural and urban) were 70,639 and 6699, respectively. At present, the district has 30 rural and 1 urban kebeles (the smallest administrative units in urban and rural areas). A total of 6 health centers and 31 Health posts were available in the District [8]_._

### Sample size determination and sampling procedures

A single population proportion formula, [*n* = (Z α/2)2 p (1-p)/d2], was used to estimate the sample size. The following assumptions were made while calculating the sample size. The *degree of precision or margin of error* chosen to be 0.05 with the reliability coefficient of 1.96 % certainly (z = 1.96). The proportion of deliveries conducted in West Gojjam Zone, Sekela District where (12.1 %) of them gave birth at health facilities while majority of them (87.9 %) delivered at home [9]. Therefore, the proportion of delivery attended by skilled birth attendants was 12.1 % (*p* = 0.121 and q = 0.879). As multistage sampling technique was used, we multiplied the calculated sample size by a design effect of 2 and added 10 % for non-response. Then the total sample size calculated was 361.

Multistage sampling method was used to select 361 women of child bearing age (15–49 years). In Dega Damot District, there are 31 kebeles (30 rural kebeles and one urban kebele). Among 31 kebeles, one urban and eight rural kebeles were selected by simple random sampling technique. The total sample size was then allocated proportionately to the number of designated kebeles. Women of child bearing age (15–49 years) who gave birth within the past 2 years and lived in Dega Damot District for a minimum of 1 year prior to the survey were included in the study. When there were more than one eligible women in the selected household, a lottery method was used to pick one of them.

### Data collection tools and procedure

#### Quantitative data

Data were collected by using a pretested and structured questionnaire administered by face to face interviews. The questionnaire was adapted from other similar studies (9–11). The questionnaire was originally developed in English; then translated into local language (Amharic) and back into English to ensure its accuracy by an expert. To ensure the quality of data, the questionnaire was pretested in 5 % of the sample size in similar set up before the actual data collection. The questionnaire consists of socio-demographic characteristics (age, ethnicity, religion, educational status and occupational status and obstetric history including women’s place of delivery for their last child birth, women’s past obstetrical history and others. Data were collected by trained 6 female health extension workers (HEW’s) who were working in Health posts.

#### Qualitative data

This study was also triangulated with a qualitative method. Semi-structured interview guide was used to collect the data. Three focus group discussions (FGDs) were conducted each involving a total of 6–8 participants. Out of the three FGDs, one was conducted in Urban district while the remaining two FGDs were conducted in rural districts. Each group consisted of women within the age range of 18–45 years who delivered at home in the past 12 months and reported for MCH clinic registration or postpartum care. The women were selected purposively in the clinics and were followed into the communities where the health facilities are located and the FGDs were held. Permission to participate in the focus group was sought prior to the meeting. The data collection team included the researcher and two female research assistants. The female research assistants were hired as data collectors in order to guarantee the clients’ openness to discussions and to conform to the study’s feminist Theoretical stances. The discussions were audio-taped using a digital voice recorder and a trained data collector took written notes. Each session lasted for 30–40 min.

The discussions were focused on different themes relating to factors preventing utilisation of health facilities for delivery care including the women’s previous experience with health facilities for labour and delivery care; attitude of health workers; perceived ANC care; and their choice of delivery place.

### Data analysis

Data were cleaned and entered into EPI INFO version 3.5.4 and were exported to SPSS version 20.0 for data analysis. The data were expressed in percentages, graphs, means and standard deviations.

The qualitative data analysis was done simultaneously with data collection. Thematic analysis was used and all data were transcribed verbatim. All FGDs were transcribed and translated into English by two investigators fluent in Amharic. Where there was no consensus, the investigators reviewed the transcriptions and original recordings until agreement was reached. The transcripts were then categorized into themes and analyzed manually. All data were stored in a lockable cupboard and were only accessible to the researchers.

## Results

### Socio-demographic characteristics

A total of 361 eligible women were included in the study yielding the response rate of 100 %. The mean and median age of the respondents was 30.9 and 30.0 respectively. One hundred seven (29.6 %) of the respondents were in the age range of 25–29 years. Majority 296 (82 %) were rural residents. As regards their marital status, about 287 (79.5 %) of them were married. Among the total study participants, majority 360 (99.7 %) were orthodox religion and all of them were Amhara by ethnicity. Two hundred fifteen (59.6 %) of them were farmers followed by house wives, 44 (12.2 %). Of the total respondents, more than half, 182 (50.4 %) were illiterate while 38 (10.5 %) of them had diploma and above. The mean monthly income of respondent’s was $ 41.6 USD [SD ± $ 30.94 USD] (Table 1).

**Table 1:** Socio-demographic characteristics of the respondents (*n* = 361) in Dega Damot District, Ethiopia, May 2014

Variables	Frequency	Percent
Place of residence		
Rural	296	82.0
Urban	65	18.0
Age of respondents		
20–24	55	15.2
25–29	107	29.6
30–34	89	24.7
35–39	71	19.7
+40	39	10.8
Mean + SD	30.93 ± 6.006	
Marital status		
Married	287	79.5
Single	7	1.9
Divorced	11	3.0
Separated	39	10.8
Widowed	17	4.7
Religion		
Orthodox	360	99.7
Protestant	1	0.3
Ethnicity		
Amhara	361	100.0
Respondent’s occupation		
House wife	44	12.2
Governmental Worker	38	10.5
Merchant	32	8.9
Farmer	215	59.6
Daily labors	16	4.4
Student	16	4.4
Husband’s occupation, *n* = 326		
Farmer	197	60.4
Daily laborer	21	6.4
Merchant	53	16.3
Governmental Worker	55	16.9
Respondent’s educational status		
Illiterate	182	50.4
Read and write	70	19.4
Primary education (1–8)	35	9.7
Secondary education	19	5.3
Certificate	17	4.7
Diploma and above	38	10.5
Husband’s educational status, *n* = 326		
Illiterate	68	20.9
Read and writes	119	36.5
Primary education (1–8)	50	15.3
Secondary education	16	4.9
Certificate	16	4.9
Diploma and above	57	17.5
Monthly house hold income (USD)		
$ <=19.95	77	21.3
$ 19.95–34.95	102	28.3
$ 35–49.95	78	21.6
$ >=50	104	28.8
Mean ± SD	41.6 ± 619.5	

### Institutional delivery use and attendants during delivery

In this study, of the total respondents only 38.2 % of women gave birth in health facilities for their most recent birth while the most 223 (61.8 %) delivered at home. Home delivery in our study is defined as a delivery that is not being attended by a trained health worker using a safe delivery kit or attended by non-trained people (the majority of whom are family members) during delivery. In our study, the proportion of women assisted by skilled birth attendants during institutional delivery was 89.1 % followed by Health extension workers (8.0 %). Skilled birth attendant is an accredited health professional such as a midwife, doctor or nurse - who has been educated and trained to proficiency in the skills needed to manage normal (uncomplicated) pregnancies, childbirth and the immediate postnatal period, and in the identification, management and referral of complications in women and newborns.

Most women (87.4 %) who did not deliver in health facilities were assisted by Lay birth attendants (families, friends or neighbors) followed by Health extension workers (7.2 %), and traditional birth attendants (5.4 %), respectively. A Lay birth attendant is a person without any training in midwifery skills who assisted a mother to give birth without delivery kit (Table 2).

**Table 2:** Percentage distribution of women who delivered in the past 1 year according to place of delivery and attendants during delivery, Dega Damot District, East Gojjam 2014

Variable	Number (*n* = 361)	Percent (100 %)
Place of delivery		
Home	223	61.8
Health facility(HF)	138	38.2
Attendant during HF delivery		
Health workers	123	89.1
Health Extension workers	11	8.0
Don’t remember	4	2.9
Attendant during home delivery		
Family/friends/neighbors	195	87.4
Health Extension workers	16	7.2
Traditional birth attendant	12	5.4

### Reasons for utilization of institutional delivery

**Fig. 1:**
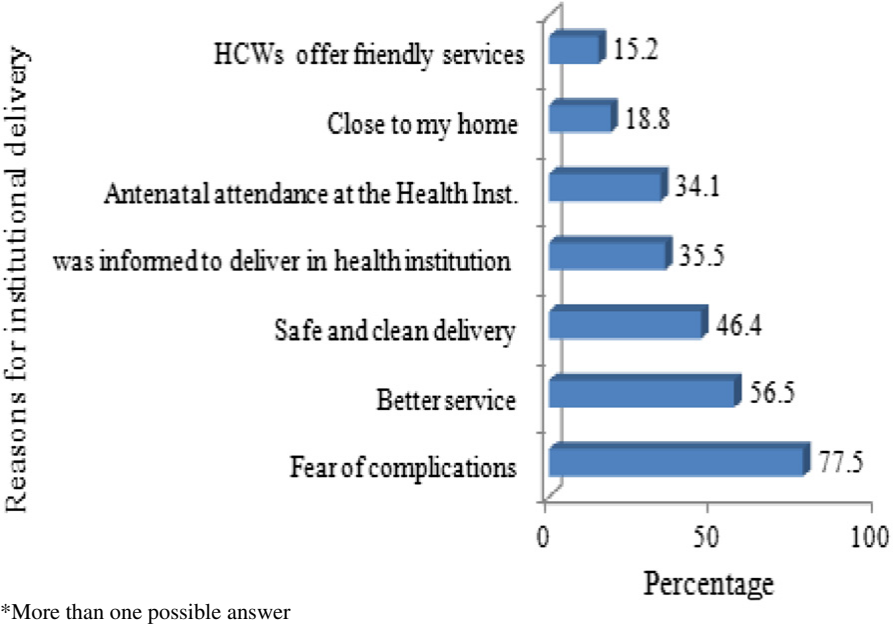
Reasons for the choice of institutional delivery (*n* = 138) in Daga Damot District, Ethiopia, May 2014

Women who delivered in health facilities were asked the reasons for doing so. Among the respondents who attended institutional delivery, the main reasons cited for selecting skilled delivery service were multiple and included fear of complications (77.5 %), to get better service (56.5 %) safe and clean delivery (46.4 %) and they were communicated to deliver at health facilities during ANC attendance, 47 (35.5 %) (Fig. 1).

The qualitative data has supported the quantitative findings. One of the FGD participants has said: “*Benefits of ANC are well- recognized in our community. For example, pregnant women who had a good knowledge and attended ANC clinic usually delivers at health institution. In addition, women attend health institution for delivery after they encountered complications such as too much bleeding and/or prolonged labor”. (A 35- year old FGD participant)*

### Past obstetric characteristics

Two hundred and seven (57.3 %) respondents reported that they were less than 18 years old during their first pregnancy. One hundred thirty five (37.4 %) had attended ANC service, of which 59 (43.7 %) had attended 2–4 visits, 41 (30.4 %) had attended one visit and 35 (25.9 %) had more than four visit during their last pregnancy (Table 3).

**Table 3:** Obstetric and maternal characteristics (*n* = 361) in Dega Damot District, Ethiopia, May 2014

Variables	Frequency	Percent
Age at first marriage		
<18 years.	207	57.3
>18 years.	154	42.7
Age at first pregnancy		
<18 years.	91	25.2
>18 years.	270	74.8
Gravidity		
1	99	27.4
2–5	202	56.0
>5	60	16.6
Parity		
1	155	42.9
2–5	186	51.5
>5	20	5.5
Antenatal care attendance		
Yes	135	37.4
No	226	62.6
No. of visits of antenatal care		
1	41	30.4
2–4	59	43.7
>4	35	25.9

Health care provider’s behavior and attitudes are also determinant factor for a choice of place of delivery for pregnant women, some of the health workers are impolite, with offensive language and refuting to support the patients, these attitudes inhibit the women to deliver in health facilities, and nevertheless, positive attitudes of health workers draw women to deliver in health facilities. For example in the present study one woman during focused group discussion said:

*“During my last few days before I delivered, I went to the ANC clinic, and the midwife advised me sympathetically that I had to go to the district hospital for delivery because my child was not in the right position. I went to the district hospital and gave birth to healthy baby”. (FGD participant, aged 32 years).*

Another experience from FGD with a mother who had given birth a month ago, gave a clear picture of a provider–client relationship. *“When I went to the health facility for delivery, I was impressed by the nurse who cared for me so much. She was so human, respectful and compassionate”* (FGD Participant, aged 28 years).

Vaginal examinations women are exposed to in labor and delivery is frequently regarded and stated as painful, recurrent and regularly quoted as a factor preventing the use of health facility for delivery. In the current study one participant shared her lived experience as stated beneath:

“*In my previous delivery I was exposed to series of vaginal examination……. Had it been my first delivery with the health facility, then I would not ever go there again”. My faith (Muslim) doesn’t let another person to insert his hands into a married woman’s genital parts (Vagina). (FGD Participant, 34 years).*

## Discussion

In Ethiopia, just like most developing countries, a number of women still prefer to deliver at home than to deliver in the health facilities. In Ethiopia, only 15 % percent of births are delivered at a health facility despite more than 40 % of pregnant women having at least one ANC visit during pregnancy. In this study, we found only 38.2 % of women gave birth in health facilities for their most recent birth. This prevalence is lower than the prevalence in studies conducted in Uganda and India [10, 11]. Nevertheless it is relatively higher compared to the national average (15 %) reported in mini Ethiopian DHS of 2014. Consequently, additional efforts are required to warrant that the Ethiopian national target of 62 % skilled delivery service is realized [12].

Several previous quantitative studies revealed that women’s use of health facility delivery services is influenced by their demographic background characteristics and their socioeconomic status. A study in rural India showed that institutional delivery is much more common for first births than for subsequent births [13]. Concerning age at delivery, another study in rural India, Punjab, revealed that institutional deliveries were more common in comparatively younger age groups, at 43 % for women age 18–25 compared with 23 % for women age 36–45 [14]. However, in our study age of the women and parity did not show any significant association with the use of health facility delivery services.

In this study, some significant factors that were found to be associated with institutional delivery were levels of education, average monthly family income, and ANC attendance. Higher levels of education and optimally attending ANC services surge the likelihood of delivering in a health facility.

Educational status of women was found to be the most significant factor influencing place of delivery. Women with secondary or higher level of education are more likely to utilize health facility delivery care services. This finding is consistent with previous studies which reported educational status of women to be the most significant associated factors for utilization of institutional delivery service [6]. This might be explained that a higher level of female education in the community leads to better awareness of the need for care during childbirth and female autonomy to utilize health facility services.

Several studies have revealed that antenatal care service utilization is a strong determinant of utilization of institutional delivery. Consistent with other study findings [9, 15–17], this study found that making one or more ANC visits is an important factor influencing delivery care service utilization. This might be advice from healthcare workers during antenatal care increases a woman’s use of institutional delivery. The healthcare workers could provide good information regarding safe health care delivery and encourage women to deliver at health facility. A previous study in Ethiopia found that proper counseling and advice to deliver at health care facility increased institutional delivery [18]. This matter was also reflected from FGD participants: *“During my last few days before I delivered, I went to the ANC clinic, and the midwife advised me sympathetically that I had to go to the district hospital for delivery because my child was not in the right position. I went to the district hospital and gave birth to healthy baby”. (FGD participant, aged 32 years).*

Another FGD participant shared her testimony about benefits of ANC attendance as stated beneath: *“Benefits of ANC are well- recognized in our community. For example, pregnant women who had a good knowledge and attended ANC clinic usually delivers at health institution. In addition, women attend health institution for delivery after they encountered complications such as too much bleeding and/or prolonged labor”. (A 35- year old FGD participant).* In our study, women and their families thought that skilled birth care is necessary only when complications occur. A qualitative study among rural women in central Nepal shows similar results that women go to the health facility only if they experience a problem during labor [19].

Stable maternal source of income was also significantly associated with institutional delivery in this study. In principle, health care in Ethiopia for pregnant women is free. However, healthcare facilities seldom run out of stock, requiring a pregnant woman to incur some expenses for supplies or services. Besides, some women must pay transportation fare to travel from home to the health facility. Therefore, pregnant women with good sources of income are better able to pay for transport and unexpected healthcare facility costs. A previous study from Ethiopia also found that women with stable income were more likely to utilize institutional delivery [20].

The present study was correspondingly consistent with the studies conducted in the Latin American and Caribbean region, and in Europe, North Africa, and the Middle East, over 50 % of all women in richest quintile reported that choosing delivery at public facilities [21].

Users of health care services often consider attitude of health workers when deciding where to seek care. Women often mentioned positive attitude of health workers as a main boosting factor for the utilization of health services particularly institutional delivery. The women reported varied witnesses. For example in the present study, a mother who had given birth to her third baby a month ago, gave a clear picture of a provider–client relationship*: “When I went to the health facility for delivery, I was impressed by the nurse who cared for me so much. She was so human, respectful and compassionate” (FGD Participant, aged 28 years).*

Expectations were commonly ruled by experience of the women which predisposed their future expectations. Women expect staff with a positive attitude. These included advice, politeness, giving reassurance, accessible by the bedside and exercising patience. Support, care and companionship throughout labor and delivery are exceedingly valued. Regrettably these were not always obtained in health facilities. As these were always assured for home deliveries women opt to deliver at home as oppose to a health facility [21].

Procedures during labor can be started with little discussion, but might be considered disgraceful or awful to women. For instance, vaginal examinations women are exposed to in labor and delivery is frequently regarded and stated as painful, frequent and often cited as a factor deterring the use of health facility for delivery. A woman who had her 2^nd^ child in the hospital narrated: “*in my previous delivery I was exposed to series of vaginal examination……. Had it been my first delivery with the health facility, then I would not ever go there again”. My faith (Muslim) doesn’t let another person to insert his hands into a married woman’s genital parts (Vagina). (FGD Participant, 34 years).*

The midwife should be able to perform vaginal examination; ensuring the woman’s own well-being. The required skills and ability of a midwife includes being able to communicate commendably, need to foster effective interpersonal communication skills and an attitude of respect for the women’s right to be a full partner in the management of their pregnancy, child birth and the post natal period [21].

There are several potential limitations to our study. First, as in all cross-sectional studies, we can infer association but not causation from our results. Second, social desirability bias could have magnified there ported rates of health facility delivery; nevertheless, the study was confidential and data collectors were instructed to guarantee women that their responses could not be related to them. Data on child birth and place of delivery were collected retrospectively from mothers so there is potential for recall bias; to minimize this, we used data on the most recent birth within the past 1 year. Third, our study was limited to 9 villages (1 urban and 8 rural) in one district and hence the findings cannot be generalized to the country or the region as a whole.

## Conclusion

This study revealed that women’s institutional delivery service utilization in the study area is low. We identified significant factors that determine utilization of institutional delivery care in rural Ethiopia. Factors promoting the choice of institutional delivery were education level, ANC attendance, and high family income. The qualitative data has described and gave an insight of the contributing factors that influence the women using the health institutions for delivery. These included: ANC attendance, positive attitude of health workers and complications during labor and delivery. The preference for a health facility delivery was largely due to the understanding that if complications occurred either during labor or delivery, this was the only place where they could be managed.

We thus recommend that the government takes the matter of female education and empowerment more earnestly besides to steady health education on the hazard of home delivering as a method of decreasing maternal mortality in the country and achieving the millennium development goals 5. Maternal health care utilization would be fortified by making services more accessible, providing training for health care personnel, mainly health extension workers in health posts, and adopting a strategy to make the services available when needed. Services friendly to women should also be encouraged in all health facilities that provide maternal health care.

## Abbreviations

ANC: Antenatal Care
CSA: Central Statistical Agency
EDHS: Ethiopian Demographic and Health Survey
FGD: Focused Group Discussion
HCW: Health Care Workers
HEW: Health Extension Workers
IRB: Institutional Review Board
MDG: Millennium Development Goals
MMR: Maternal Mortality Ratio
